# Draft Genome Sequence of *Sphingomonas* sp. WG, a Welan Gum-Producing Strain

**DOI:** 10.1128/genomeA.01709-15

**Published:** 2016-02-11

**Authors:** Hui Li, Zhi-mei Feng, Ya-jie Sun, Wan-long Zhou, Xue Jiao, Hu Zhu

**Affiliations:** Centre for Bioengineering and Biotechnology, China University of Petroleum (East China), Qingdao, People’s Republic of China

## Abstract

We report the draft genome sequence of *Sphingomonas* sp. WG, a high welan gum-producing strain with a yield of 33 g/L. The core of *wel* cluster for welan gum biosynthesis contained 24 coding sequences in the genome, which will provide the genetic information on welan gum production.

## GENOME ANNOUNCEMENT

Recently, the organisms of the genus *Sphingomonas* have attracted more attention because they have great potential to be applied in many biotechnological applications, such as degradation of environmental pollutants, bioremediation, and wastewater treatment. At the same time, they are also an important microbial resource for the biopolymer synthesis*.* The produced biopolymers, sphingans, are bacterial exopolysaccharides sharing a similar backbone of tetrasaccharide repeating units containing two glucose, one glucuronic acid, and one rhamnose or mannose (1). Welan gum, as an important sphingan, exhibits excellent rheological properties and good stability over a broad range of pH (2 to 12) and temperatures (up to 150°C) (2). *Sphingomonas* sp. WG is a high welan gum-producing strain screened from sea-mud samples by our laboratory and preserved in the China Center for Type Culture Collection under the number M2013161 (3). The highest yield of welan gum reached 33 g/L, which was much higher than the yield (26.3 g/L) reported by Li et al. (4). The whole-genome sequencing of *Sphingomonas* sp. WG might provide more information on the biosynthetic mechanism of welan gum by revealing possible genes involved in its biosynthesis.

Therefore, the genome of *Sphingomonas* sp. WG was sequenced using a paired-end Illumina HiSeq 2500 system. A total of 17,749,410 high-quality paired-end reads 100 bp in length were obtained. *De novo* assembly was performed using Velvet version 1/2/10 (5) and resulted in 107 contigs with a contig *N*_50_ of 78.6 kb. Next, these contigs were further assembled into 31 scaffolds using SSPACE basic version 2.0 (6). The scaffold *N*_50_ was approximately 237.5 kb, and the largest scaffold assembled spans approximately 570.92 kb. The total length of the draft genome was 4,042,222 bp with a mean GC content of 65.9%. Gene prediction and annotation were performed using Glimmer version 3.02, the Rapid Annotation using Subsystem Technology (RAST) annotation server, and the NCBI Prokaryotic Genomes Automatic Annotation Pipeline (7–9). A total of 3,857 protein-coding sequences and 46 structural RNAs were predicted. The structural RNAs mainly included 43 tRNAs, one 5S RNA, one large subunit rRNA, and one small subunit rRNA. Compared to genome sequences available at RAST, the multiple sequence alignment showed that *Sphingopyxis alaskensis* RB2256 (score, 551), *Sphingobium japonicum* UT26S (score, 501), and *Sphingomonas wittichii* RW1 (score, 487) are the closest neighbors of *Sphingomonas* sp. WG.

The *wel* cluster for welan gum biosynthesis was predicted in the genome of *Sphingomonas* sp. WG. The core area of *wel* cluster is about 24 kb and contains about 24 genes, mainly covering the four-gene *rml* cluster *rmlABCD*, the genes (*welB*, *welK*, *welL*, and *welQ*) encoding glycosyltransferases in the assembly of the tetrasaccharide repeating units, and some enzyme-encoding genes (like *welS*, *welG*, *welC*, and *welE*) that participated in the polymerization, chain-length regulation, and export of welan gum. Other genes involved in the synthesis of UDP-glucose, UDP-D-glucuronic acid, and GDP-mannose were dispersed on the genome. All these genes may play important roles in the biosynthesis of welan gum. The annotated cluster is very similar to the *wel* cluster predicted by Schmid et al. (10) based on the genome of *Sphingomonas* sp. ATCC 31555 (11).

### Nucleotide sequence accession numbers.

This whole-genome shotgun project has been deposited at DDBJ/EMBL/GenBank under the accession number LNOS00000000. The version described in this paper is the first version, LNOS00000000.
